# The activity and stability of CeO_2_@CaO catalysts for the production of biodiesel†

**DOI:** 10.1039/c8ra06884d

**Published:** 2018-09-24

**Authors:** Ni Zhang, Huiyuan Xue, Rongrong Hu

**Affiliations:** Key Laboratory of Applied Surface and Colloid Chemistry, School of Chemistry & Chemical Engineering, Shaanxi Normal University Xi'an 710119 China rrhu@snnu.edu.cn

## Abstract

A novel CeO_2_@CaO catalyst was prepared *via* a hydrothermal method. The physicochemical properties and morphologies of the prepared CeO_2_@CaO catalysts were characterized by X-ray diffraction, N_2_ physisorption, CO_2_ temperature-programmed desorption, X-ray photoelectron spectroscopy, transmission electron microscopy and energy dispersive X-ray analysis. It was found that the prepared CeO_2_@CaO catalyst had a distinct core–shell structure. The catalytic activity of the CeO_2_@CaO sample as a heterogeneous catalyst for the transesterification of soybean oil to produce biodiesel has been studied. The results showed that the optimum yield of biodiesel can reach 98% over the CeO_2_@CaO-60 catalyst under the reaction conditions of 3 wt% catalyst, methanol to oil molar ratio of 6 : 1, reaction temperature of 70 °C and reaction time of 6 h. Stability tests indicated that the biodiesel yield can reach more than 80% even after 9 reaction cycles due to the strong synergic interaction between CaO and CeO_2_.

## Abstract 2

The stability test results for the CaO, CeO_2_@CaO-60 and CeO_2_–CaO-60 catalysts for biodiesel production.

## Graphical abstract

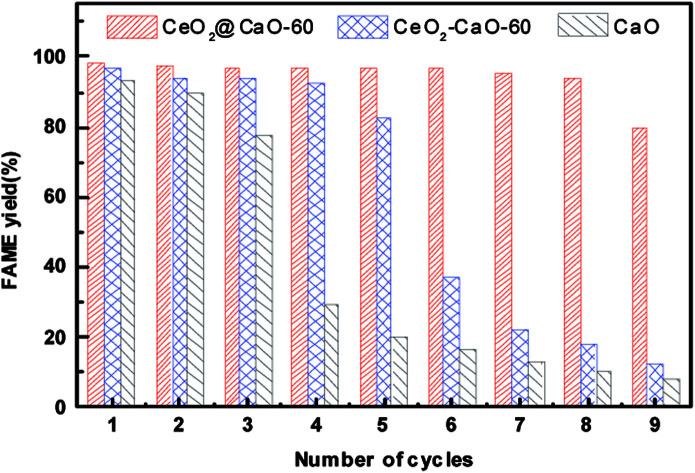

## Introduction

As is well known, due to the shortage of fossil fuels, environmental pollution and ecological deterioration, people have turned their attention to low-carbon, environmentally friendly, clean and safe renewable resources. Biodiesel has received increasing attention in recent years since it is renewable, biodegradable, non-toxic and harmless to humans and environments.^1,2^ Its raw materials mainly include oil crops, plants,^3^ animal fats^4^ and catering waste oil,^5^ which are low cost and readily available. Biodiesel is of high calorific value, has a stable combustion performance and can be used in compression-ignition engines. Thus it is a very promising alternative to conventional diesel.

There are many ways to produce biodiesel. Biodiesel produced by the direct blending method and the microemulsion method^6,7^ doesn't meet the diesel standards. While biodiesel prepared by the esterification and transesterification method^8^ has very similar properties to petrochemical diesel, so it can be directly used in diesel engines. Now, homogeneous catalytic transesterification becomes a major industry production process for biodiesel production by using trifluoroacetic acid, sulfuric acid, sodium hydroxide, or potassium hydroxide.^9^ However, homogeneous catalytic transesterification^10^ has several disadvantages. For example, the catalyst is difficult to reuse, and a large amount of wastewater could be generated in the homogeneous catalytic process. In contrast, heterogeneous catalytic transesterification copes with most of the shortcomings of homogeneous process, such as easily separated, reusable, and free from saponification reactions. Yee^11^ prepared Al_2_O_3_/Zr(SO_4_)_2_ catalyst for biodiesel production and the yield of biodiesel reached 90.32%. Ma^12^ synthesized KOH/γ-Al_2_O_3_ catalyst and applied it to the transesterification of rapeseed oil. The yield of biodiesel reached 84.52% at 60 °C for 1 h with a methanol to oil molar ratio of 9 : 1. Bimetallic Au@Ag nanoparticles showed high catalytic activities for the transesterification reaction and the highest yield of biodiesel from sunflower oil was about 86.9%.^13^ Magnetic materials,^14^ metallic monolithic catalysts^15^ and nanocatalysts such as TiO_2_ (ref. 16) and ZnO^17^ had also shown potential applications in biodiesel production.

In general, heterogeneous solid base catalysts have better catalytic activity than solid acid catalysts^18^ for feedstock oils with a low acid value, which is of higher catalytic efficiency and lower cost.^19,20^ Nowadays, a large number of different heterogeneous basic catalysts^21^ such as hydrotalcite, layered-structured minerals, zeolites and alkaline earth metal oxides have been tested for biodiesel production. It has been reported that alkaline earth metal oxides were capable of producing higher biodiesel yield because of its higher basicity and the number of highly basic sites at the edges of the metal oxide cluster is one of the key factors to affect catalytic performance in the transesterification reaction.^22^ Among them, CaO, as a promising catalyst, has drawn much attention in transesterification reactions for biodiesel production. CaO shows high catalytic activity and does not have any major negative impact on the environment.^23^ It also has great economic advantages because of its low price and the convenience to be obtained from natural and waste materials. However, CaO is sensitive to the free fatty acids (FFAs). During the transesterification reactions, the leached calcium species will react with FFAs and result in soap formation. The deactivation problem caused by leaching of Ca^2+^ has been a main drawback of CaO catalyst.^22^ In order to improve the stability of calcium, numerous researchers have attempted to modify CaO with the second metal oxide, use Perovskites containing Ca, or support CaO onto carriers. Significant enhancement on the reusability has been achieved over these CaO-based catalysts in the transesterification reaction due to the high surface area, strong basicity, and reduced sensitivity to FFAs.^24^ So far, CaO–CeO_2_ catalyst has also been studied and it showed great potential compared with other CaO-based catalysts in the transesterification reaction. Wong^25^ prepared the CaO–CeO_2_ catalysts *via* a impregnation method and the highest biodiesel yield reached 95%. Yu^26^ reported the production of biodiesel over the CaO–CeO_2_ catalysts by transesterification of *Pistacia chinensis* oil with methanol. The optimum yield of 91% was achieved at 110 °C for 6 h with a methanol to oil molar ratio of 30 : 1. Yan^27^ synthesized the CaO–CeO_2_/HAP catalysts which presented excellent performance and stability due to the low leaching of catalyst components in the product phase. Reyero^15^ used CaO–CeO_2_ supported metallic monolithic catalysts for the production of biodiesel and the highest conversion of sunflower oil was about 99%, though significant leaching of the active catalytic layer was found during the second reaction cycle. Ceria itself was found inactive in the transesterification reaction,^22,28^ however, the synergy between calcium oxide and cerium oxide could reduce the leaching of CaO in biodiesel products when calcium oxide was incorporated into the cerium oxide.

In this study, a kind of novel core–shell CeO_2_@CaO catalyst was prepared and tested in the transesterification of soybean oil with methanol under mild reaction conditions. The physicochemical properties of the prepared CeO_2_@CaO catalysts were characterized by using several analytic techniques. Effects of the surface area, basicity, CaO loadings and morphology of the core–shell materials on biodiesel yield were studied. Catalyst stability and recycling performance in transesterification reaction were also investigated and the possibility of its reuse in repeated batch reactions was estimated.

## Experimental

### Materials

Calcium nitrate (Ca(NO_3_)_2_·4H_2_O), cerium chloride heptahydrate (CeCl_3_·7H_2_O), urea (CO(NH_2_)_2_), methanol (CH_3_OH), hexadecyl trimethyl ammonium bromide (CTAB), calcium oxide (CaO) and cerium oxide (CeO_2_) were purchased from Sinopharm Chemical Reagent Factory, of analytical reagent grade. Non-transgenic soybean oil (Jiusan Cereals & Oils Industry Group Co., Ltd.) was purchased from the local market.

### Catalysts preparation

The core–shell CeO_2_@CaO catalysts with composition 20–60 wt% CaO were prepared by a hydrothermal method. To prepare the core–shell CeO_2_@CaO sample with a CaO content of 20 wt%, 0.21 g of CaO and 0.1 g CTAB were added to 60 mL deionized water and dispersed under ultrasonication for 10 min. Then 0.86 g CeCl_3_·7H_2_O and 3 g CO(NH_2_)_2_ were added and dissolved in the suspension completely followed by stirring for 0.5 h at room temperature. The mixture was then transferred to 100 mL Teflon-lined stainless steel autoclave and heated in an oven at 90 °C for 24 h. After that, the products were separated by centrifugation and washed with anhydrous ethanol. Followed by a dry process at 100 °C for 3 h and then calcined at 750 °C for 6 h in air, the core–shell CeO_2_@CaO sample with a CaO content of 20 wt% (CeO_2_@CaO-20) was obtained. The other core–shell CeO_2_@CaO catalysts having 40 wt% and 60 wt% CaO contents could be prepared following this procedure and they were labeled as CeO_2_@CaO-*x*, where *x* represented the amount of CaO loaded on the catalyst.

The CeO_2_–CaO catalysts with composition 20–60 wt% CaO were prepared *via* wet impregnation method and labeled as CeO_2_–CaO-*x*, where *x* represented the amount of CaO loaded on the catalyst. Briefly, 1 g of Ca(NO_3_)_2_·4H_2_O was dissolved in 20 mL deionized water and a complementary amount of CeO_2_ was added slowly into this solution followed by heated at 90 °C until the water in solution completely evaporated. Then the resulting powder was dried in an oven at 100 °C for 3 h and calcined in a muffle furnace at 750 °C for 6 h. Finally, the CeO_2_–CaO-*x* catalyst was obtained.

### Catalysts characterization

X-ray powder diffraction (XRD) was carried out on a Bruker D8 ADVANCE diffractometer fitted with Cu–K radiation to determine the phase identity of the synthesized samples. The specific surface area of each sample was measured by using a Micromeritics ASAP 2460 Surface Area and Porosity Analyzer with the BET method. Transmission electron microscopy (TEM) images were examined by using a JEOL model JEM 2010 EX instrument. Energy dispersive X-ray analysis (EDAX) was recorded on a FEI Tecnai G2F20 instrument and operated at an accelerating voltage of 200 kV. Surface electronic states were analyzed using X-ray photoelectron spectroscopy (XPS) with an AXIS ULTRA spectrometer. The amount of catalyst elements was determined using the inductively coupled plasma optical emission spectrometer (ICP-OES), Perkin-Elmer Optima 3000V. Furthermore, the basic properties of samples were determined using temperature-programmed desorption with CO_2_ as a probe molecule, which were performed on a Micromeritics AutoChem 2920 II instrument with the temperature-programmed mode. The amount of CO_2_ desorbed in the temperature range of 100–900 °C was detected by thermal conductivity detector.

### Transesterification reaction

The transesterification reaction of soybean oil and methanol was carried out in a three necked glass reactor with a reflux condenser and a magnetic stirrer. Typically, the calcined catalyst (3 wt% calculated with respect to soybean oil) and the soybean oil were added in 30 mL methanol under stirring with a constant speed for the transesterification reactions. The methanol to soybean oil molar ratio was varied from 1 : 1 to 14 : 1. The mixture was refluxed at 60–80 °C in an oil bath with constant magnetic stirring 1–6 h. After the reaction finished, the catalysts were then separated by centrifuging at 3000 rpm for 30 min. The obtained liquid products were placed in a funnel for phase separation. Biodiesel floated on the top layer while glycerol at the bottom. The desirable biodiesel obtained was further purified by distillation to remove the excess methanol.

The composition of the product obtained was analyzed by Gas Chromatography-Mass Spectrometry (GC-MS: 6890 N GC/5973 MS, Agilent Technologies). Since the main component of biodiesel is fatty acid methyl ester (FAME), the FAME yield also could be determined by ^1^H nuclear magnetic resonance (^1^H NMR).^29^^1^H NMR spectra of the purified biodiesel production were recorded at ambient temperature on a Varian VXR-400 MHz spectrometer using standard procedures. The chemical shifts were referenced to the residual peaks of CHCl_3_ in CDCl_3_ (7.26 ppm). The percent yield of FAME was calculated by the ratio of the area of the single peak associated with methyl esters at 3.53 ppm and the peak at 2.20 ppm representative of the α-methylene protons in the ester molecule.^29,30^

## Results and discussion

### Catalysts characterization

**Fig. 1:**
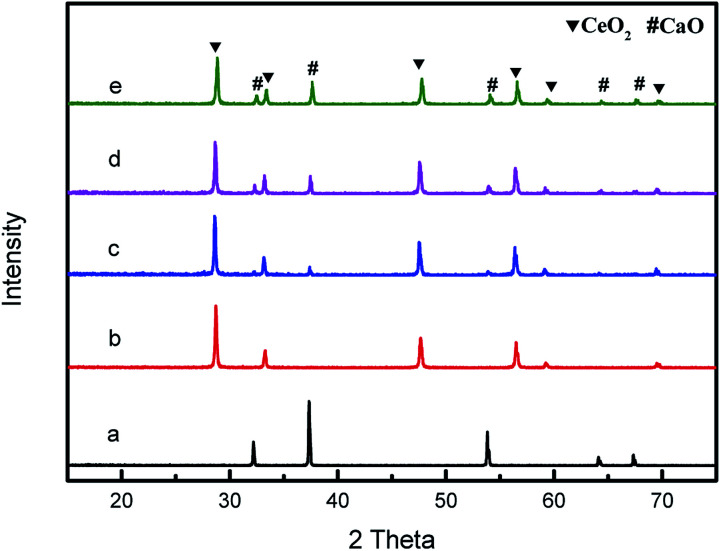
XRD patterns of the CeO_2_@CaO catalysts (a): CaO, (b): CeO_2_, (c): CeO_2_@CaO-20, (d): CeO_2_@CaO-40, (e): CeO_2_@CaO-60.

The power XRD patterns for various compositions CeO_2_@CaO catalysts are shown in Fig. 1. For all of the samples, the diffraction peaks were clear and showed similar patterns. The peaks at 28.68°, 33.30°, 47.85°, 56.35° and 59.8° were ascribed to the (111), (200), (220), (311) and (222) planes of pure CeO_2_ with the fluorite-type cubic structure. The pure CaO gave well-defined diffraction peaks at 2*θ* values of 32.45, 37.64, 53.88, 64.41 and 67.68. The XRD analysis of these CeO_2_@CaO catalysts revealed they had the separate CaO and CeO_2_ crystalline phases and no new species or a binary phase such as CaCeO_3_ was found over these samples. From Fig. 1, the increase in CaO content resulted in an increase and a slight decrease in the peak intensities of CaO and CeO_2_, respectively. This may be attributed to the coating of Ca^2+^ species on the surface of ceria and their higher X-ray scattering factor of Ca^2+^ compared to the Ce^4+^ ions.^28^

The surface areas, pore volumes and average pore diameters of the CeO_2_@CaO catalysts are listed in Table 1. As the CaO content in catalysts increased from 20 wt% to 60 wt%, the surface areas and pore volumes of the samples increased significantly from 8.75 m^2^ g^−1^ to 16.44 m^2^ g^−1^ and 0.064 cm^3^ g^−1^ to 0.103 cm^3^ g^−1^, respectively. While further increasing calcium loading from 60 wt% to 100 wt%, the surface area of the catalysts decreased from 16.44 m^2^ g^−1^ to 11.65 m^2^ g^−1^. From Table 1, the pore diameters of all samples were in the range of 10–50 nm, which is beneficial for the reaction of large reactants because the limitation of pore diffusion in the transesterification reaction can be reduced using mesopore catalysts.^31^

**Table 1:** Surface area, pore volume and average pore diameter of the CeO_2_@CaO catalysts

Samples	Surface area m^2^ g^−1^	Pore volume cm^3^ g^−1^	Average pore diameter nm
CeO_2_@CaO-20	8.75	0.0640	19.26
CeO_2_@CaO-40	13.56	0.0809	27.37
CeO_2_@CaO-60	16.44	0.1038	26.30
CaO	11.65	0.1155	20.90

**Fig. 2:**
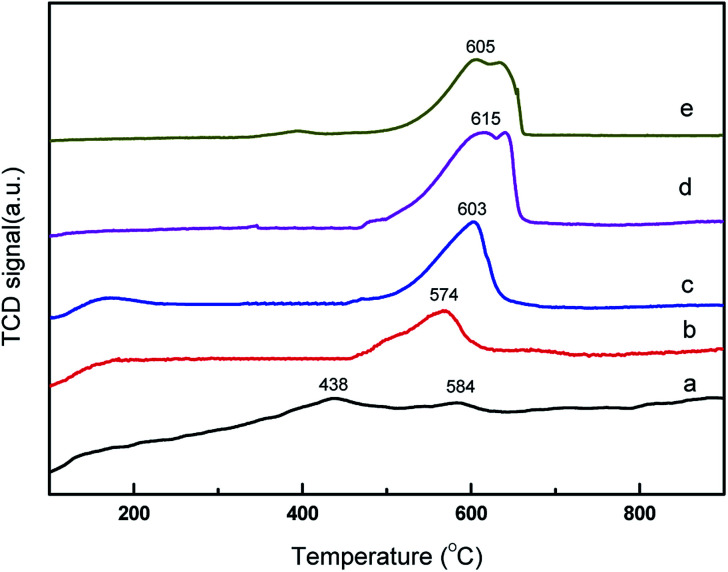
CO_2_-TPD analysis of the CeO_2_@CaO catalysts (a): CeO_2_, (b): CeO_2_@CaO-20, (c): CeO_2_@CaO-40, (d): CeO_2_@CaO-60, (e): CaO.

The basic property of the catalysts was evaluated using temperature programmed desorption of CO_2_. CO_2_-TPD profile over the core–shell CeO_2_@CaO catalysts is shown in Fig. 2. It revealed that some of the CeO_2_@CaO catalysts contained two desorption peaks. The desorption peaks around 200 °C can be assigned to the interaction between CO_2_ and weak basic sites. The desorption peaks around 570–640 °C can be assigned to existence of strong basic sites. The strong basic sites of CeO_2_@CaO catalysts showed the existence of oxygen in Ca–O, Ce–O_2_ ion pairs and isolated O_2_^−^ anions, which was helpful to initiate the transesterification reaction.^32^ From Fig. 2, the basicity of the CeO_2_@CaO-60 catalyst was found to be higher than both bulk CaO and CeO_2_. The improved basicity of the sample was due to the synergetic effect between CaO and CeO_2_. Furthermore, it was found that the CeO_2_@CaO-60 sample had the highest CO_2_ desorption temperature and largest number of basic sites, which also had been proved it had the highest catalytic activity in the transesterification reaction.

**Fig. 3:**
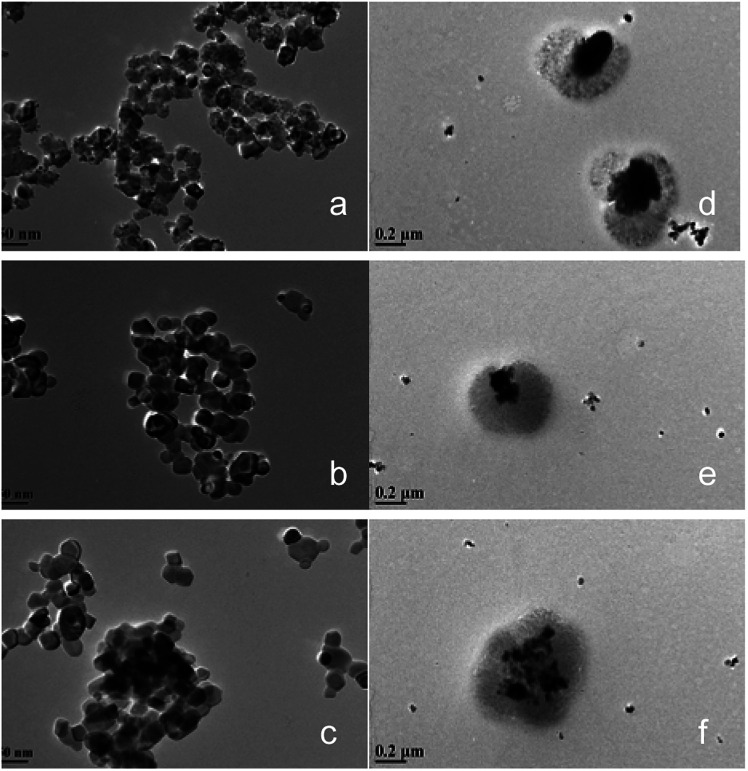
TEM images of the CeO_2_@CaO and CeO_2_–CaO catalysts (a): CeO_2_–CaO-20, (b): CeO_2_–CaO-40, (c): CeO_2_–CaO-60, (d): CeO_2_@CaO-20, (e): CeO_2_@CaO-40, (f): CeO_2_@CaO-60.

Fig. 3 shows the transmission electron microscope (TEM) images of the CeO_2_@CaO and CeO_2_–CaO catalysts. It was noted that the morphology of CeO_2_@CaO samples was very different from that of CeO_2_–CaO. The existence of the dark areas (inner layer, core) and bright areas (outer layer, shell) in TEM images clearly shows the core–shell structure of CeO_2_@CaO nanocomposite with a particle size of 400–600 nm, in which the core is spatially and compactly encaged within a shell. The diameter of the core and shell was 100–300 nm and 200–400 nm, respectively. The particle size of CeO_2_–CaO catalysts was much smaller than that of CeO_2_@CaO and it was in the range of 15–40 nm for all the CeO_2_–CaO catalysts. In addition, it can be seen that with the increase of Ca contents, the particle size of the catalysts increased. The results indicated that the Ca content had a significant effect on the particle size of the catalysts.

**Fig. 4:**
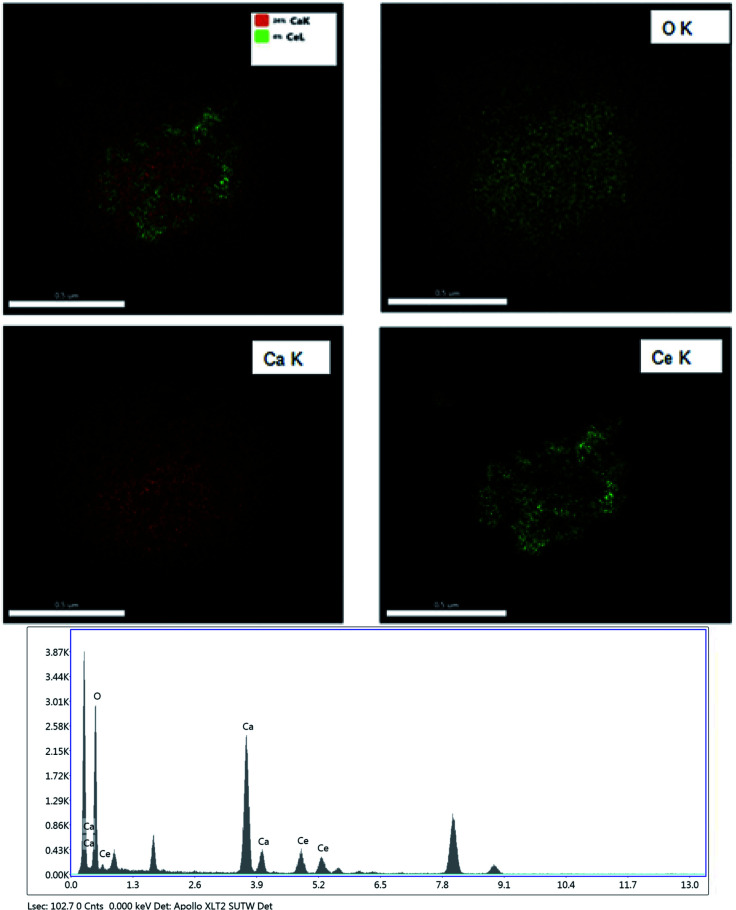
The EDS mapping and EDX analysis of the CeO_2_@CaO-60 catalyst.

Fig. 4 shows the EDS mapping of the CeO_2_@CaO-60 catalysts. It demonstrated just only Ce, Ca, and O elements existed in the core–shell samples and it was CaO that covers the core of CeO_2_ completely. The elemental compositions of the CeO_2_@CaO-60 catalyst also could be estimated by using Energy dispersive X-ray analysis (EDXA) and the results are listed in Table 2. For the CeO_2_@CaO-60 catalyst, the CaO content of that is 60 wt% and the theoretical Ca/Ce atomic ratio is about 4.61 by calculation. From Table 2, the measured Ca/Ce atomic ratio is 4.01, which means the predicted and experimental compositions were in good agreement with each other with no significant deviation observed.

**Table 2:** The content of elements obtained from EDX analysis for the CeO_2_@CaO-60 catalyst

Element	Weight(%)	Atomic(%)
O K	24.32	54.46
Ca K	40.85	36.61
Ce L	34.83	8.93

**Fig. 5:**
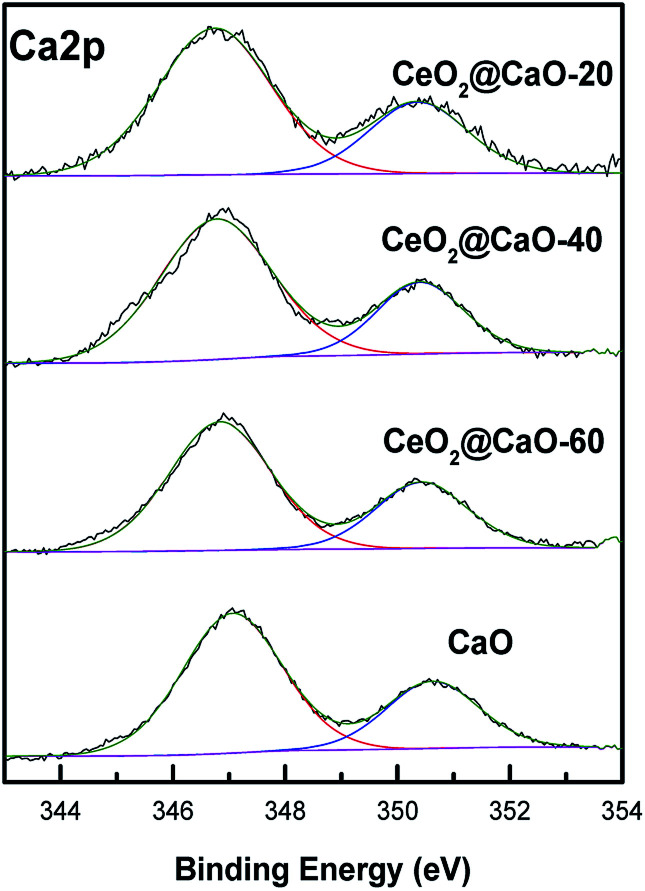
Ca 2p XPS spectra of the CeO_2_@CaO catalysts.

**Fig. 6:**
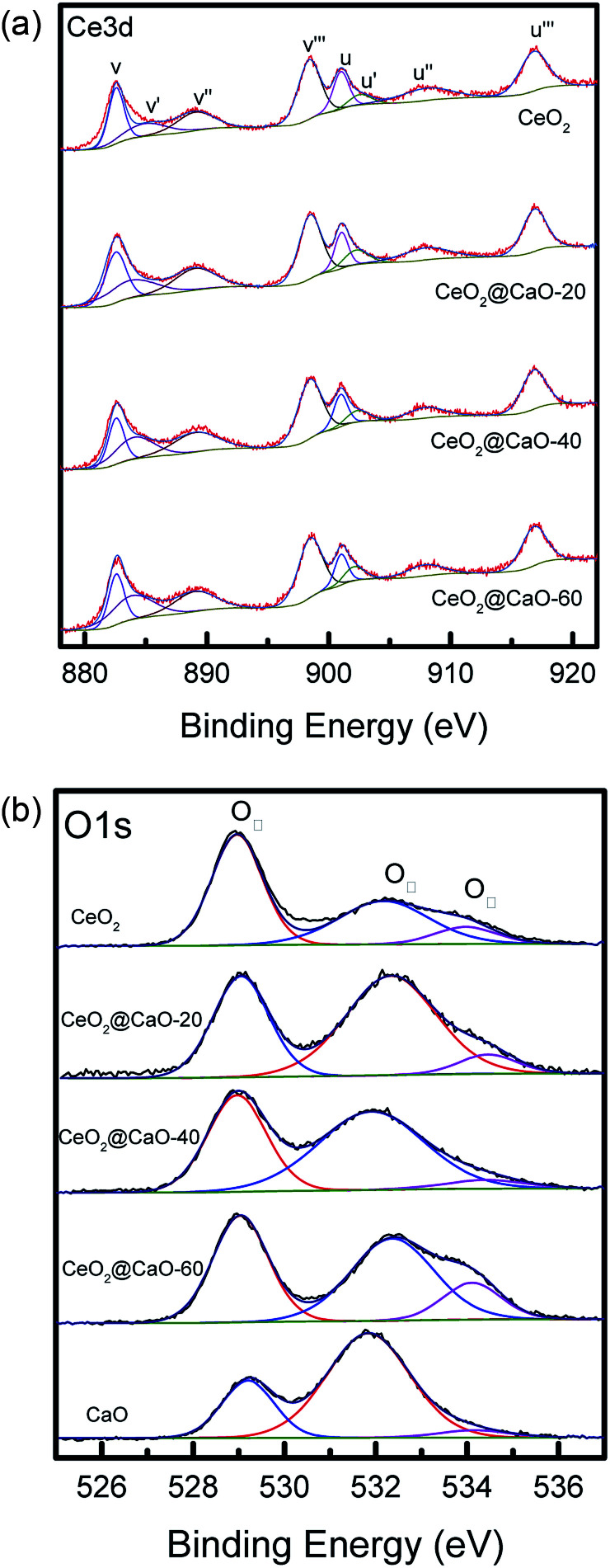
Ce 3d XPS spectra (a) and O 1s XPS spectra (b) of the CeO_2_@CaO catalysts.

Fig. 5 and 6 present the XPS spectra of Ca 2p, Ce 3d and O 1s for CeO_2_@CaO samples, respectively. It can be observed that Ca 2p spectra (Fig. 5) displayed two main characteristic peaks with BE around at 347 eV and 351 eV. With the increase of Ce content, the BE of Ca 2p shifted slightly from 347.2 eV toward a lower value 346.6 eV, suggesting the interaction of Ce with the catalyst surface.

The Ce 3d level has a very complicated structure and the Ce 3d spectra can be deconvoluted into eight peaks: v (∼883.8 eV), v′ (∼885.6 eV), v′′ (∼888.2 eV), v′′′ (∼898.1 eV), u (∼901.7 eV), u′ (∼906.0 eV), u′′ (∼908.2 eV) and u′′′ (∼916.0 eV). The four U bands represent Ce 3d_3/2_, and the four V bands represent Ce 3d_5/2_. Six peaks corresponding to three pairs of spin–orbit doublets [(V, U), (V′′, U′′), and (V′′′, U′′′)] can be identified with the 3d^10^ 4f^0^ state of the Ce^4+^ species, while two peaks due to one pair of doublets (V′, U′) characterize the 3d^10^ 4f^1^ state of the Ce^3+^ species.^33^ As shown in Fig. 6a and Table 3, when the Ca content increased, the BE of Ce^3+^ and Ce^4+^ slightly shifted to a higher value, suggesting that the electron transfer from lattice oxygen atoms to metal atoms.^22^ In addition, the surface atomic ratio of Ce^3+^ : Ce^4+^, which was calculated by all the peaks, decreased with the Ca content, indicating a strong interaction between Ca and Ce.^34^

**Table 3:** XPS results of the CeO_2_@CaO catalysts

Catalyst	Ca 2p_3/2_	Ce 3d_5/2_		Ce^3+^ : Ce^4+^	O_I_ : O_II_
		(Ce^4+^)	(Ce^3+^)		
CeO_2_		898.1	883.9	0.49	1.43
CeO_2_@CaO-20	346.6	898.1	884.0	0.45	0.62
CeO_2_@CaO-40	346.8	898.3	884.2	0.41	0.69
CeO_2_@CaO-60	347.0	898.4	884.8	0.34	0.86
CaO	347.2				0.35

The O 1s spectra of these samples (Fig. 6b) showed three states of surface oxygen: the lattice oxygen O_I_ (∼529.0 eV), the adsorbed oxygen O_II_ (∼531.0 eV) and the adsorbed carbonates and/or water O_III_ (∼533.5 eV).^35^ The ratio of O_I_ to O_III_ for all samples was calculated in Table 3 (See ESI† for the details about the XPS results of O 1s for the CeO_2_@CaO samples). From it, the O_I_ to O_II_ ratios of the CeO_2_@CaO catalysts are remarkably higher than that of the CaO catalyst, indicating that the incorporation of cerium can increase the amount of lattice oxygen on the surface of the CeO_2_@CaO catalysts due to the synergistic effect between CaO and CeO_2_.^33^ The O^2−^ species has been reported to be the strong base site for solid base catalysts.^36,37^ For the CeO_2_@CaO-60 catalyst, the O_I_ to O_II_ ratios is 0.86, which is highest among all the CaO-based catalysts, so it has the strongest basic strength. It is particularly noted that the CeO_2_ had very low basicity due to the nature of the oxygen species of CeO_2_ (ref. 38), though it also has a high O_I_ to O_II_ ratio.

### Catalytic activity

**Fig. 7:**
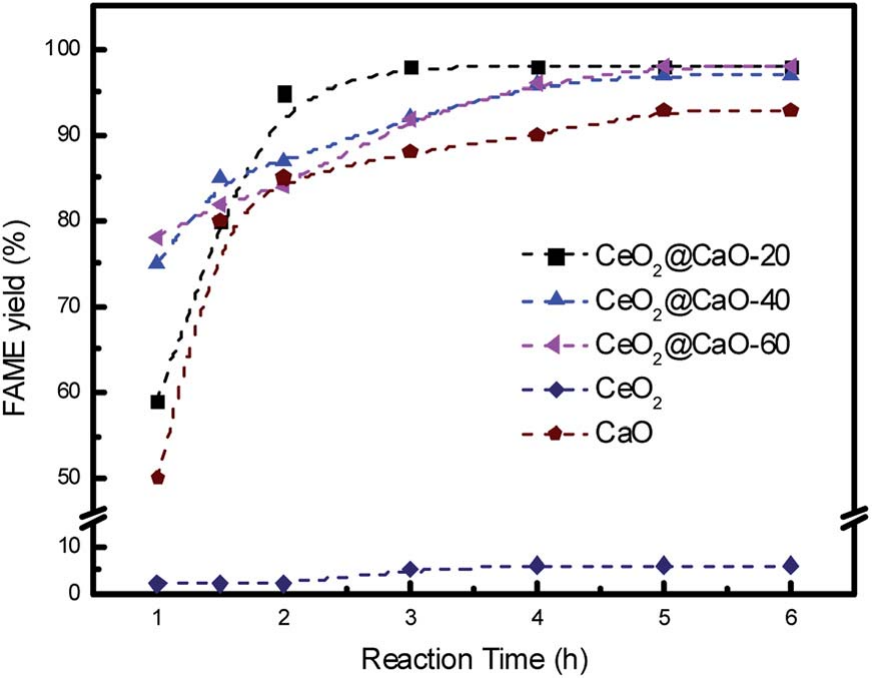
Effect of reaction time on the FAME yield over the CeO_2_@CaO catalysts.

Catalytic activity of various CeO_2_@CaO catalysts for the transesterification of soybean oil was carried out under conditions of 3 wt% catalysts, methanol to soybean oil molar ratio of 12 : 1 and reaction temperature of 70 °C. Fig. 7 shows the effect of reaction time on FAME yield over all samples. From it, the cerium oxide catalyst showed very poor catalytic performance due to the low basicity and the FAME yield was less than 10% even after 6 h. For all the CeO_2_@CaO and pure CaO catalysts, the transesterification reaction started rapidly and the FAME yield reached more than 80% in the first 2 h. The maximum yield was 98% with a reaction time of 3 h over CeO_2_@CaO-20 catalyst and after this time the FAME yield has been constant, indicating that the active sites of the catalyst were available for the transesterification reaction.

**Fig. 8:**
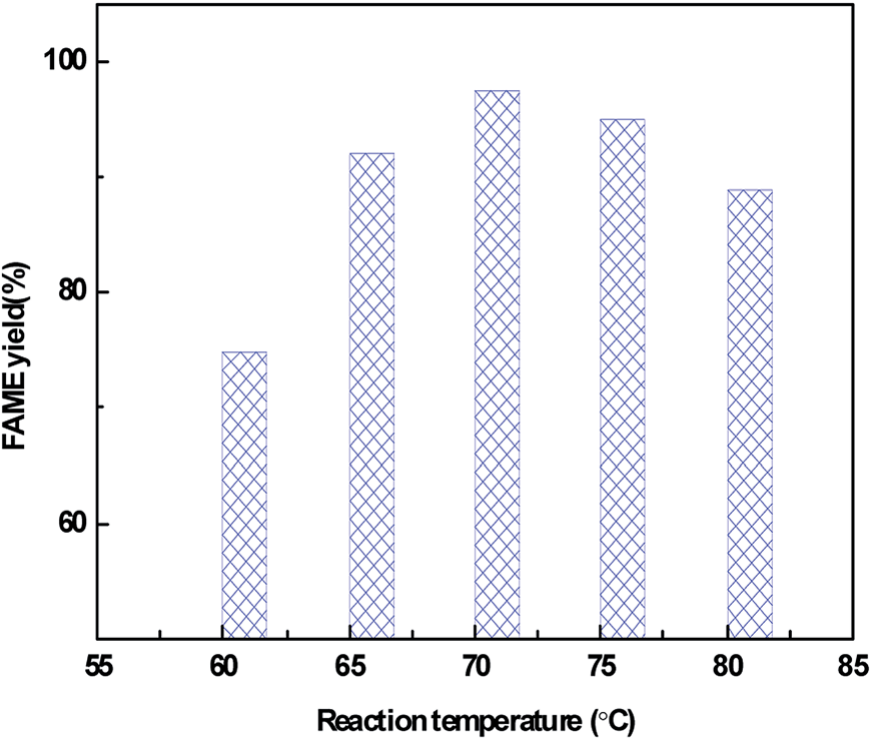
Effect of reaction temperature on the FAME yield over the CeO_2_@CaO-60 catalyst.

The effect of reaction temperature on the catalytic activity over the CeO_2_@CaO-60 catalyst with a methanol to oil molar ratio of 12, catalyst amount of 3% and reaction time of 6 h is shown in Fig. 8. From it, the FAME yield can reach more than 70% from 60 °C to 80 °C and the highest yield is 98% at 70 °C. Below 70 °C, the FAME yield increased with an increase in the reaction temperature and decreased when further increasing the temperature. The higher temperature is favourable for biodiesel synthesis since the transesterification reaction is an endothermic reaction. However, when the reaction temperature was higher than 64.7 °C, a large amount of methanol evaporation rose with increasing temperature, resulting in a concentration decrease of methanol in the reaction. These two combined effects leaded to an optimum reaction temperature of 70 °C for the transesterification reaction.

**Fig. 9:**
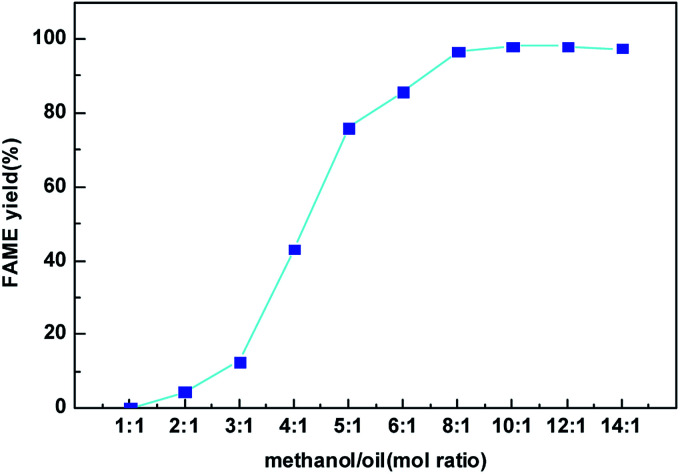
Effect of methanol to oil mol ratio on the FAME yield over the CeO_2_@CaO-60 catalyst.

Fig. 9 presents the effect of the methanol to oil molar ratio on the FAME yield at the best condition found in Fig. 8. Molar ratio of methanol to oil is one of the most significant factors affecting the FAME yield as well biodiesel production cost. Since the transesterification reaction is reversible, higher molar ratios are beneficial to increasing the oil conversion by shifting this equilibrium to the production of biodiesel. From Fig. 9, when methanol to oil molar ratio increased from 1 : 1 to 10 : 1, the FAME yield catalyzed by the CeO_2_@CaO-60 sample increased gradually and reached the maximum values of 98%. However, the FAME yield was slightly reduced when the methanol to oil molar ratio was 14 : 1. The decrease in FAME yield might be due partly to the remaining of glycerol in the biodiesel phase since methanol could act as an emulsifier^27,28^ and render glycerol separation complicated.

### Catalyst stability

As mentioned earlier, one of the advantages of the heterogeneous catalytic transesterification over the homogeneous process is the easy separation and reuse of the solid catalyst.

**Fig. 10:**
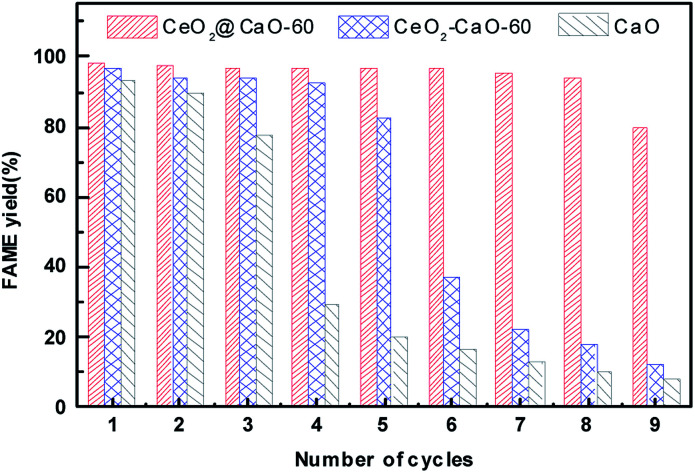
The stability test of the CaO, CeO_2_@CaO-60 and CeO_2_–CaO-60 catalysts.

Fig. 10 presents the stability study of the pure CaO, CeO_2_@CaO-60 and CeO_2_–CaO-60 catalyst for the transesterification of soybean oil at 70 °C for 6 h with a methanol to oil molar ratio of 12 : 1 and catalyst amount of 3 wt%. After each reaction finished, the catalyst was separated, washed with a mixture solution of methanol and *n*-heptane for several times and then dried in oven for 6 h before used in the next cycle. From Fig. 10, the FAME yield decreased sharply over pure CaO and was less than 30% at the fourth cycle. The CeO_2_–CaO-60 catalyst had better stability than CaO though the FAME yield eventually dropped 50% after the sixth run. For the CeO_2_@CaO-60 catalyst, it could maintain more than 80% FAME yield even after 9 cycles and has better performance than both pure CaO and the CeO_2_–CaO-60 catalyst. In general, there are two possible factors that contribute to the deactivation of the CaO based catalysts. One is the leaching of CaO into product phase and the other is the surface poisoning such as the adsorption of fatty acid, glycerol or glycerides on the active sites.

**Fig. 11:**
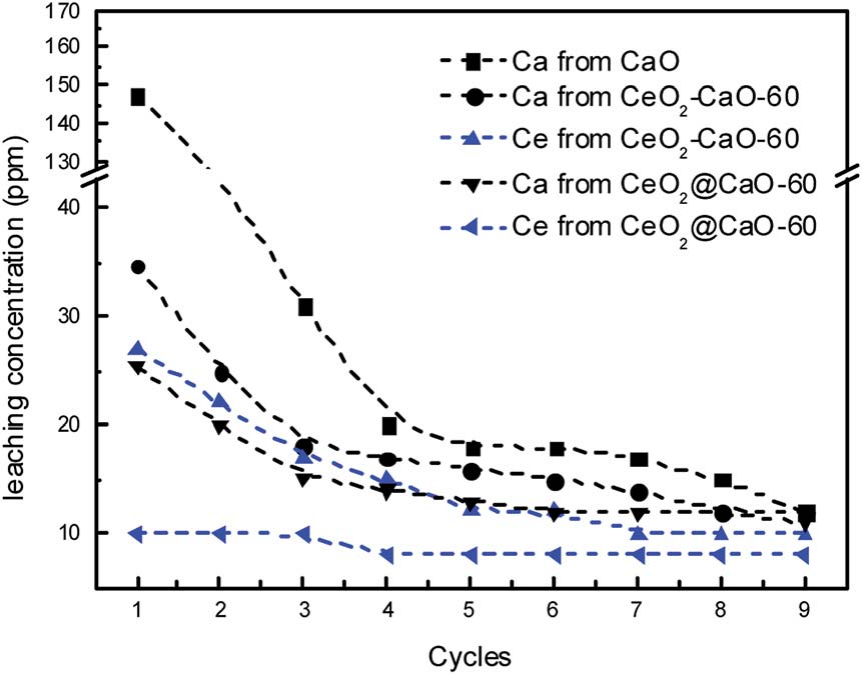
The leaching concentrations of calcium and cerium species during the stability test.

In order to investigate the leaching of those used catalysts, the concentrations of Ca and Ce species were measured using ICP method after each cycle and presented in Fig. 11. The results revealed an obvious loss of calcium species in the biodiesel products over all the three catalysts. The pure CaO showed the highest concentration of calcium in the biodiesel layer with 147.4 ppm detected. In contrast, the dissolved calcium species in the biodiesel phase catalyzed by the CeO_2_@CaO-60 and CeO_2_–CaO-60 samples were about 25.3 and 34.7 ppm, respectively. The calcium concentration of those catalysts leached into the product phase decreased sharply at the first three cycles, becoming lower than 15 ppm after the 9th cycle. It is interesting to note that the leaching of Ce was more than 25 ppm over the CeO_2_–CaO-60 catalyst. This implies that the deactivation of this catalyst is due to the leaching of Ce and Ca. However, for the CeO_2_@CaO-60 catalyst, the loss of Ce in the biodiesel was in the range of 8–10 ppm, which was relatively small. This result indicated that special core–shell structure of the CeO_2_@CaO catalyst could inhibit the leaching of Ce into the product phase when it was embedded in calcium oxide. CeO_2_, in turn, is able to stabilize the active phases and improve the stability of the catalyst.

## Conclusions

The CeO_2_@CaO catalysts, which have been successfully prepared *via* a hydrothermal method, showed better catalytic performance than pure CaO and the CeO_2_–CaO catalysts synthesized by a wet impregnation method for the production of biodiesel from soybean oil. The highest FAME yield can reach 98% over the CeO_2_@CaO-60 catalysts under the optimum reaction conditions. In addition, the catalyst could be reused up to 9 times with good activity and get more than 80% FAME yield. The excellent performance of the CeO_2_@CaO catalysts for the transesterification reaction was possibly due to the strong synergic interaction between CeO_2_ and CaO. This interaction was attributed to the special core–shell structure of these samples with Ce embedded in calcium oxide. CeO_2_ could stabilize the active phases, reduce the leaching of Ca into the product phase and improve the stability of the catalyst during the reactions. These results suggest that a core–shell CeO_2_@CaO material is a promising catalyst for the green biodiesel production process.

## Conflicts of interest

There are no conflicts to declare.

## Supplementary Material

RA-008-C8RA06884D-s001

## References

[cit1] Luque R., Lovett J. C., Datta B., Clancy J., Campelo J. M., Romero A. A. (2010). Energy Environ. Sci..

[cit2] Falasca S. L., Flores N., Lamas M. C., Carballo S. M., Anschau A. (2010). Int. J. Hydrogen Energy.

[cit3] Dizge N., Aydiner C., Imer D. Y., Bayramoglu M., Tanriseven A., Keskinlera B. (2009). Bioresour. Technol..

[cit4] Punsuvon V., Nokkaew R., Somkliang P., Tapanwong M., Karnasuta S. (2015). Energy Sources, Part A.

[cit5] Ngo T. A., Kim M. S., Sim S. J. (2011). Int. J. Hydrogen Energy.

[cit6] Jain S., Sharma M. P. (2010). Renewable Sustainable Energy Rev..

[cit7] Tong K., Zhao C. H., Sun Z. C., Sun D. J. (2015). ACS Sustainable Chem. Eng..

[cit8] Tao G. J., Hua Z. L., Gao Z., Chen Y., Wang L. J., He Q. J., Chen H. R., Shi J. L. (2012). RSC Adv..

[cit9] Miao X. L., Li R. X., Yao H. Y. (2009). Energy Convers. Manage..

[cit10] Su C. H. (2013). Appl. Energy.

[cit11] Yee K. F., Lee K. T., Ceccato R., Abdullah A. Z. (2011). Bioresour. Technol..

[cit12] Ma H. B., Li S. F., Wang B. Y., Wang R. H., Tian S. J. (2008). J. Am. Oil Chem. Soc..

[cit13] Banerjee M., Dey B., Talukdar J., Kalita M. C. (2014). Energy.

[cit14] Zhang P. B., Shi M., Liu Y. L., Fan M. M., Jiang P. P., Dong Y. M. (2016). Fuel.

[cit15] Reyero I., Moral A., Bimbela F., Radosevic J., Sanz O., Montes M., Gandia L. M. (2016). Fuel.

[cit16] Salinas D., Araya P., Guerrero S. (2012). Appl. Catal., B.

[cit17] Yang Z. Q., Xie W. L. (2007). Fuel Process. Technol..

[cit18] Yang K. L., Huang S., Pan H., Zhang H., Liu X. F., Yang S. (2017). RSC Adv..

[cit19] Gong S. W., Lu J., Wang H. H., Liu L. J., Zhang Q. (2014). Appl. Energy.

[cit20] Fu J. Y., Chen L. G., Lv P. M., Yang L. M., Yuan Z. H. (2015). Fuel.

[cit21] Babajide O., Musyoka N., Petrik L., Ameer F. (2012). Catal. Today.

[cit22] Thitsartarn W., Kawi S. (2011). Green Chem..

[cit23] Marinkovic D. M., Stankovic M. V., Velickovic A. V., Avramovic J. M., Miladinovic M. R., Stamenkovic O. O., Veljkovic V. B., Jovanovic D. M. (2016). Renewable Sustainable Energy Rev..

[cit24] Granados M. L., Poves M. D. Z., Alonso D. M., Mariscal R., Galisteo F. C., Moreno-Tost R., Santamaria J., Fierro J. L. G. (2007). Appl. Catal., B.

[cit25] Wong Y. C., Tan Y. P., Taufiq-Yap Y. H., Ramli I., Tee H. S. (2015). Fuel.

[cit26] Yu X. H., Wen Z., Li H. L., Tu S. T., Yan J. Y. (2011). Fuel.

[cit27] Yan B. B., Zhang Y., Chen G. Y., Shan R., Ma W. C., Liu C. Y. (2016). Energy Convers. Manage..

[cit28] Teo S. H., Rashid U., Taufiq-Yap Y. H. (2014). RSC Adv..

[cit29] Shah M., Ali S., Tariq M., Khalid N., Ahmad F., Khan M. A. (2014). Fuel.

[cit30] Ren Y. B., He B. Q., Yan F., Wang H., Cheng Y., Lin L. G., Feng Y. H., Li J. X. (2012). Bioresour. Technol..

[cit31] López D. E., Goodwin J. G., Bruce D. A., Lotero E. (2005). Appl. Catal., A.

[cit32] Lee A. F., Bennett J. A., Manayil J. C., Wilson K. (2014). Chem. Soc. Rev..

[cit33] Liu B., Li C. M., Zhang G. Q., Yan L. F., Li Z. (2017). New J. Chem..

[cit34] Qin F., Nohair B., Shen W., Xu H. L., Kaliaguine S. (2016). Catal. Lett..

[cit35] Xiang X. M., Zhao H. H., Yang J., Zhao J., Yan L., Song H. L., Chou L. J. (2016). Appl. Catal., A.

[cit36] Wen Z. Z., Yu X. H., Tu S. T., Yan J. Y., Dahlquist E. (2010). Bioresour. Technol..

[cit37] Fraile J. M., Garcia N., Mayoral J. A., Pires E., Roldan L. (2010). Appl. Catal., A.

[cit38] Russbueldt B. M. E., Hoelderich W. F. (2010). J. Catal..

