# Supplementary material for: The activity and stability of CeO2@CaO catalysts for the production of biodiesel
Source: RSC Adv. 2018 Sep 24;8(57):32922–9. doi: 10.1039/c8ra06884d (PMC9086313; doi:10.1039/c8ra06884d)
Supplement: RA-008-C8RA06884D-s001 [file RA-008-C8RA06884D-s001.pdf]

Electronic Supplementary Material (ESI) for RSC Advances.
This journal is © The Royal Society of Chemistry 2018

Supporting Information

The activity and stability of CeO_2@CaO catalysts for the production of biodiesel

Ni Zhang, Huiyuan Xue, Rongrong Hu*,

Key Laboratory of Applied Surface and Colloid Chemistry, School of Chemistry & Chemical Engineering, Shaanxi Normal University, Xi'an, 710119, China.

* To whom correspondence should be addressed. rrhu@snnu.edu.cn

TABLE CAPTIONS

Table S1. XPS results of O 1s for the CeO_2@CaO catalysts

Table S1 XPS results of O 1s for the CeO_2@CaO catalysts

Catalyst	Binding energy (eV)			Surface percentage (%)		
	O_{I}	O_{II}	O_{III}	O_{I}	O_{II}	O_{III}
CeO_2	528.5	531.6	533.2	53.03	37.05	9.92
CeO_2@CaO-20	528.6	531.2	534.2	35.87	57.1	7.03
CeO_2@CaO-40	528.6	531.3	533.6	38.6	56.57	4.83
CeO_2@CaO-60	528.7	531.7	533.3	39.66	46	14.34
CaO	529.6	531.3	533.6	24.56	71.12	4.32
